# Low concentrations of monosodium glutamate (MSG) are safe in male *Drosophila melanogaster*

**DOI:** 10.1186/s13104-018-3775-x

**Published:** 2018-09-17

**Authors:** Keneth Iceland Kasozi, Sarah Namubiru, Oliver Kiconco, Hellen Wambui Kinyi, Fred Ssempijja, Joseph Obiezu Chukwujekwu Ezeonwumelu, Herbert Izo Ninsiima, Alfred Omachonu Okpanachi

**Affiliations:** 1 Department of Physiology, Faculty of Biomedical Sciences, Kampala International University Western Campus, Box 71, Bushenyi, Uganda; 2 College of Veterinary Medicine Animal Resources and Biosecurity, Makerere University, Box 7062, Kampala, Uganda; 3 Department of Clinical and Biopharmacy, School of Pharmacy, Kampala International University Western Campus, Box 71, Bushenyi, Uganda; 4 Department of Biochemistry, Faculty of Biomedical Sciences, Kampala International University Western Campus, Box 71, Bushenyi, Uganda; 5 Department of Anatomy, Faculty of Biomedical Sciences, Kampala International University Western Campus, Box 71, Bushenyi, Uganda

**Keywords:** *Drosophila melanogaster*, MSG Safety, MSG toxicity, Catalase activity

## Abstract

**Objective:**

Monosodium glutamate (MSG) has been marred by a lot of controversy on its safety. In a majority of experimental studies, administration of the compound has been parenteral, and yet little is known about MSG safety consumed as a food supplement. In this study, we assessed the effects of low concentrations of MSG on the activity of hydrogen scavenging, catalase activity and climbing as well as lifespan in male *Drosophila melanogaster* over a 30 days period since this has been sparsely studied.

**Results:**

No significant differences were associated with MSG at 5%, 1%, 0.2%, 0.04% on hydrogen peroxide scavenging, negative geotaxis and lifespan in *W*^*1118*^ male *D. melanogaster*. Significant differences were found in 5% MSG on catalase activity, showing that high MSG concentrations would affect tissue health in male *D. melanogaster*. MSG consumed as a food supplement would be safe at concentrations below 5% MSG.

## Introduction

Monosodium glutamate (MSG) is a common household food seasoning and its use has stirred a lot of controversy [1], although it has already received approval by international food regulatory agencies [2–4]. In a majority of experimental studies, MSG has been associated with oxidative stress [5], although these findings have been difficult to reproduce [6], thus fueling the controversy. This is because MSG is a salt of glutamate which is important in the Krebs pathway [7]. In addition, glutamate is common to foods eaten daily and its readily absorbed in the gut [8]. Furthermore, epidemiological studies have failed to identify the causative effects of the side-effects claimed to be associated with MSG [9, 10], showing a need to gain clear insight on its safety. A study by Abolaji [11] has associated low MSG concentrations in *Drosophila melanogaster* with toxicities due to its high oxidative stress, and reduced lifespan effects in a 5 days study, however, information over longer durations of exposure is limited. Bearing in mind that *D. melanogaster* have an established toxicological advantage due to their large population numbers and short lifecycle [12, 13], there was a need to examine MSG exposure in *D. melanogaster* over a longer period. In addition, oxidative stress in *D. melanogaster* has been found to decrease with an increase in age [14], showing the role of the experimental period in toxicological evaluation of MSG. The objective of the study was to determine effects of MSG supplementation on hydrogen peroxide scavenging activity, catalase activity, negative geotaxis, and lifespan in male *D. melanogaster*.

## Main text

### Methods

Monosodium glutamate (99%) was acquired from Ajinomoto Co. Inc, (Tokyo) Japan through the Institute for Innovation. Fly food was prepared in line with manufacturer recommendations [15]. In brief, 4 food types were prepared by supplementing regular fly food with MSG in the following concentration, i.e. 5% w/w MSG [4, 16], 1% w/w MSG and 0.2% w/w MSG [11], and 0.04% w/w MSG, and a control group. *Drosophila melanogaster W*^*1118*^ strains were acquired from the Bloomington Fly Stock Center, USA and these were cultured to build sufficient colonies at the Institute of Biomedical Research, Kampala International University Western Campus. From the stock population, virgin females and young males were placed on fresh food and allowed to mate. Fresh eggs were collected, and dated to ensure that the dates of birth are synchronized for all flies. In each experimental group, five replicas were created, with each vial consisting of 10 flies (i.e. n = 5 × 10 = 50), for each experiment. For all experiments, N = 50 flies × 5 groups × 4 experimental groups = 1000 flies. Flies were allowed to feed for a period of 30 days.

#### Hydrogen scavenging and catalase activity determination

*Drosophila melanogaster* flies from different experimental groups were anesthetized on ice and homogenized in 0.1 M phosphate buffer, pH 7.0 (1 mg: 10 μL). Samples were further processed as previously described [11]. After centrifugation at 4000×*g* for 10 min, the supernatant was placed into sterile Eppendorf tubes and used for biochemical experimentation at the end of the experimental period.

Catalase activity was determined by measuring the decrease in the absorbance of H_2_O_2_ (ε = 39.4 mM^−1^ cm^−1^) at 320 nm after a reaction mixture containing 500 µL of 50 mM phosphate buffer (pH 7.0), 180 µL of 300 mM H_2_O_2_, and 20 µL of sample was made to react for 2 min [11]. The decrease in absorbance of H_2_O_2_ (ε = 39.4 mM^−1^ cm^−1^) at 320 nm was recorded 2 min after a reaction mixture containing 500 μL of 50 mM phosphate buffer (pH 7.0), 180 μL of 300 mM H_2_O_2_, and 20 μL of sample. Catalase activity was then measured and expressed as micromoles of H_2_O_2_ decomposed per minute per milligram of protein. Furthermore total protein was also determined [17].

The ability of the *Drosophila* to scavenge hydrogen peroxide was determined according to the well-documented method [18] with minor modifications. In brief, 40 mM of H_2_O_2_ were prepared in phosphate buffer at a pH 7.4. To improve on the concentration of H_2_O_2_ from the supernatant of *D. melanogaster*, centrifugation was repeated at 16,000×*g* and the refined supernatant (10 µg/mL) were mixed with 0.6 mL of 40 mM H_2_O_2_. Absorbance was taken at 320 nm after 10 min against a blank solution containing phosphate buffer [19]. A total of 10 flies were used in each vial, and these were kept in 5 replicas for each experimental group (n = 5). The percentage of hydrogen peroxide scavenging of both *D. melanogaster* and standard was determined using the equation;

$$\% {\text{ scavenging }}\left[ {{\text{H}}_{ 2} {\text{O}}_{ 2} } \right] = \left[ {\left( {{\text{A}}_{\text{c}} {-}{\text{ A}}_{\text{s}} } \right)/{\text{A}}_{\text{c}} } \right] \, \times { 1}00;$$

where A_c_ is the absorbance of the control and A_s_ is the absorbance in the presence of the sample and standards.

#### Locomotion and lifespan determination

Locomotion determination was done using the negative geotaxis experiment as described by Niveditha [14] at the end of the MSG feed exposure period. A transparent plastic ring tube which would hold 10 flies at once of standard length, 25 cm was secured on a tripod stand. In brief, the tube was tapped gently to allow the flies to settle at the bottom and a timer was started, at the moment when they were allowed to climb. The total number of flies that moved above the 12 cm mark in 20 s were counted. This was repeated for all replicas in each experimental group and the mean number was taken as the number of flies which performed positively in the experiment, and these were expressed as a percentage for each group.

Lifespan study was conducted for male fly cultures at 25 °C, 60–65% humidity and a 12 h light/12 h dark cycle in a climate-controlled incubator and the number of dead flies were counted every 3 days for a period of 30 days [20, 21].

#### Statistical analysis

Data was analyzed with Graph Pad prism 6, and presented as graphs. Factorial analysis of variance (ANOVA) was performed to test the interactions among treatment dosages using a Tukey’s multiple comparison test for all experiments, expect the lifespan data which was analyzed using Kaplan-Meier survival analysis and Mantel-Cox was performed on the survival curves, with significance being reported when *P *<* 0.05*.

### Results

**Fig. 1:**
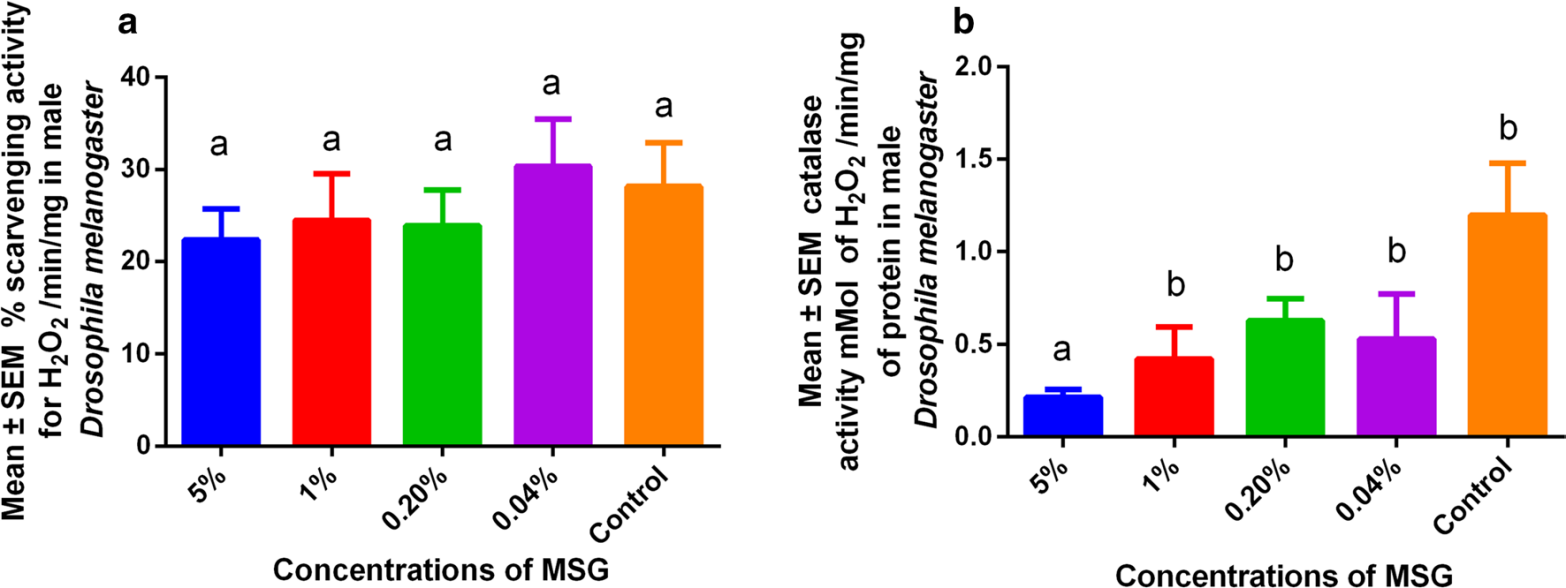
Effects of MSG in male *Drosophila melanogaster*. **a** *S*cavenging activity *of* hydrogen peroxide. **b** Catalase activity

The ability for *Drosophila* to scavenge hydrogen peroxide was low during different MSG exposure concentrations (P > 0.05) as shown in Fig. 1a. In addition, catalase activity was found to be lowest in the 5% MSG exposed male flies (P < 0.05) in comparison to the control as shown in Fig. 1b.

**Fig. 2:**
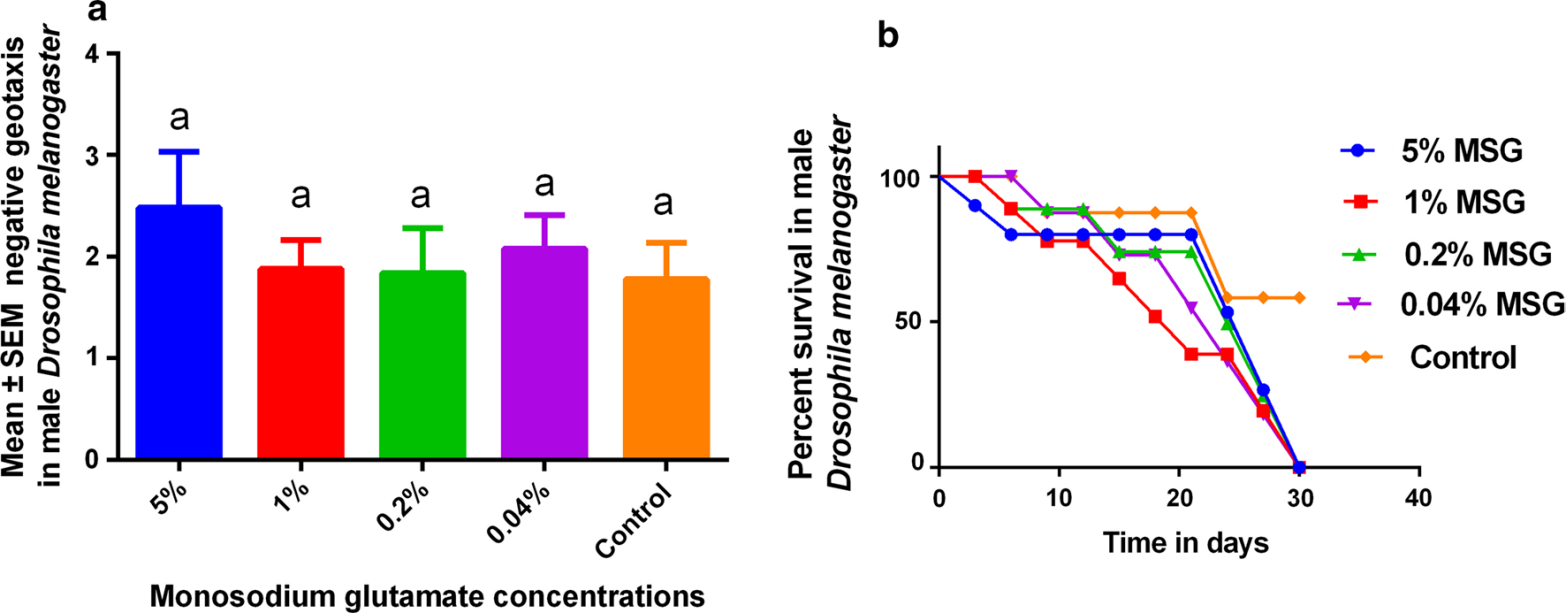
Negative geotaxis and lifespan in male *Drosophila melanogaster*

There were no significant changes in the geotaxis (Fig. 2a) although 5% MSG had a higher climbing index. In addition, survival was higher in the 0.2% and 0.04% MSG in comparison to 5% and 1% MSG (Fig. 2b). No significant differences were associated with lifespan, negative geotaxis and scavenging activity during this experimental period (Table 1).

**Table 1:** Multiple comparisons between experimental groups for negative geotaxis, catalase, scavenging and lifespan activity in male *Drosophila melanogaster*

Tukey’s multiple comparisons test	Number of flies per group (N)	Negative geotaxis	Catalase activity	Scavenging activity	Lifespan
		Adjusted *P* values			
5% vs. 1%	50	0.8305	0.9396	0.9973	Log-rank (Mantel-Cox) test for curve comparisons; *X*^*2*^ (4) = 3.644, *P *= 0.4563
5% vs. 0.2%	50	0.7950	0.5285	0.9992	
5% vs. 0.04%	50	0.9554	0.7355	0.7180	
5% vs. control	50	0.7368	0.0088*	0.8915	
1% vs. 0.2%	50	> 0.9999	0.9427	> 0.9999	
1% vs. 0.04%	50	0.9967	0.9943	0.8845	
1% vs. control	50	0.9998	0.0706	0.9773	
0.2% vs. 0.04%	50	0.9933	0.9952	0.8476	
0.2% vs. control	50	> 0.9999	0.2581	0.9626	
0.04% vs. control	50	0.9843	0.1165	0.9967	

*P values* acquired from one-way ANOVA, “*” represents significant difference

### Discussion

Low levels of MSG had no significant changes on hydrogen peroxide (Fig. 1a), which is key in the formation of reactive oxygen species [11]. These observations show that MSG at low concentrations would have enhanced antioxidant activity (Fig. 1b), demonstrating the safety of MSG once consumed in food due to its beneficial effects, which is in agreement with previous findings [22]. Bearing in mind that domestic usage of MSG has been internationally recommended [1–4]. The increased oxidative stress associated with lower concentrations of MSG [6], would be difficult to reproduce once its administered parenteral and at high concentrations as reported in several experimental studies [5]. Furthermore, adaptive responses associated with MSG at concentrations used by Abolaji [11] were not reproducible, showing the relevance of the current study.

The study also showed that low concentrations of MSG did not significantly affect the climbing index (Fig. 2a). This was important since MSG contains glutamate which is readily absorbed in the gut and assimilated into the Krebs cycle during energy production [7, 8]. Increased energy production would subsequently be associated with increased glucose breakdown, and production of more reactive oxygen species [23], leading to a decrease in antioxidant activity (Fig. 1b). Finally, lifespan was not affected by MSG at low concentrations used in the study (Fig. 2b), showing that MSG was safe in *D. melanogaster*. These findings show that effects of MSG on lifespan would be due to the high concentrations (i.e. 10%–20%) with are above safe concentrations recommended for humans [24], showing the wisdom of using small concentrations in foods. The lack of significant effects (Table 1) at these concentrations would offer a basis as to why no epidemiological data has been able to associate MSG successful with patient outcomes since humans, often consume the compound in very small concentrations, showing a need for a clinical study.

## Limitations

More information would be acquired by including more antioxidants and oxidative stress markers as we were unable to investigate them, however, this study provides basic information on the safety of low concentrations of MSG. Furthermore, information from female *D. melanogaster* would offer further insights on any toxicological effects which may be gender related.

## Abbreviations

H_2_O_2_: hydrogen peroxide
mM: millimoles
MSG: monosodium glutamate
w/w: weight by weight
μL: microliters
